# Multi‐institution consensus paper for acquisition of portable chest radiographs through glass barriers

**DOI:** 10.1002/acm2.13330

**Published:** 2021-07-02

**Authors:** Sarah E. McKenney, John M. S. Wait, Virgil N. Cooper, Amirh M. Johnson, Jia Wang, Ann N. Leung, Jessica Clements

**Affiliations:** ^1^ Department of Environmental Health and Safety Stanford University Stanford CA USA; ^2^ Medical Imaging Technology and Informatics Department Southern California Permanente Medical Group Pasadena CA USA; ^3^ Clinical Technology Department Kaiser Permanente Northern California Berkeley CA USA; ^4^ Rad/Thoracic Imaging Department Stanford University Stanford CA USA

**Keywords:** chest X‐ray, COVID‐19, infection prevention, radiation safety

## Abstract

**Background:**

To conserve personal protective equipment (PPE) and reduce exposure to potentially infected COVID‐19 patients, several Californian facilities independently implemented a method of acquiring portable chest radiographs through glass barriers that was originally developed by the University of Washington.

**Methods:**

This work quantifies the transmission of radiation through a glass barrier using six radiographic systems at five facilities. Patient entrance air kerma (EAK) and effective dose were estimated both with and without the glass barrier. Beam penetrability and resulting exposure index (EI) and deviation index (DI) were measured and used to adjust the tube current‐time product (mAs) for glass barriers. Because of beam hardening, the contrast‐to‐noise ratio (CNR) was measured with image quality phantoms to ensure diagnostic integrity. Finally, scatter surveys were performed to assess staff radiation exposure both inside and outside the exam room.

**Results:**

The glass barriers attenuated a mean of 61% of the normal X‐ray beams. When the mAs was increased to match EI values, there was no discernible degradation of image quality as determined by the CNR. This was corroborated with subjective assessments of image quality by chest radiologists. The glass‐hardened beams acted as a filter for low energy X‐rays, and some facilities observed slight changes in patient effective doses. There was scattering from both the phantoms and the glass barriers within the room.

**Conclusions:**

Glass barriers require an approximate 2.5 times increase in beam intensity, with all other technique factors held constant. Further refinements are necessary for increased source‐to‐image distance and beam quality in order to adequately match EI values. This does not result in a significant increase in the radiation dose delivered to the patient. The use of lead aprons, mobile shields, and increased distance from scattering sources should be employed where practicable in order to keep staff radiation doses as low as reasonably achievable.

## 1 INTRODUCTION

The ongoing COVID‐19 pandemic has necessitated adoption and strict adherence to infection control protocols resulting in major modifications to conventional medical practice. There is renewed interest in imaging patients with a potentially highly infectious disease via portable chest radiographs acquired through glass barriers^1^ to assess potentially COVID‐19 positive patients for probability and severity of illness.^2^ Imaging through glass barriers is motivated by efforts to conserve personal protective equipment (PPE) and reduce staff and equipment exposure.^3^

The method of acquiring portable chest radiographs through glass barriers at the University of Washington was circulated widely during the COVID‐19 pandemic^3^ and adopted by several institutions.^4^, ^5^, ^6^, ^7^ This method involves leaving the portable X‐ray units outside of the patient rooms and imaging through glass barriers, such as windows or sliding glass doors. The portable X‐ray units remain uncontaminated, thereby decreasing the use of PPE, cleaning supplies, and extra cleaning time by the staff.

The interposition of a glass barrier between the patient and the X‐ray tube during portable chest radiography acts as additional filtration of the beam and creates an additional scattering source. The additional filtration hardens the beam (increases its effective energy), potentially reducing the low‐contrast detectability due to diminished inherent “subject” contrast from the photoelectric effect. Filtration also reduces beam intensity, resulting in increased electronic noise in the images due to fewer photons. The additional scattering source may potentially lead to increased X‐ray exposure to staff and other patients within the ED at the time of the exam.

Due to these changes in imaging conditions, diagnostic medical physicists and radiation safety professionals can add value in helping to ensure safe and effective portable chest X‐ray imaging through glass barriers by largely answering three questions: (1) what are the effects on image quality? (2) What are the radiation safety implications of the additional scattering source created by the glass barrier? (3) And what technique adjustments need to be made?

These questions were independently posed to three different groups of medical physicists in California, who conducted separate investigative studies. The three groups include Kaiser Permanente Northern California (KPNC), Southern California Permanente Medical Group (SCPMG), and Stanford Health Care (SHC). Upon discovery of the similar nature of their work, the groups decided to pool their analyses to provide composite results and recommendations for dissemination. The product of this collaboration is presented in this article.

## 2 METHODS

The methods in this manuscript are divided into investigations of (1) glass barrier transmission, (2) beam penetrability and diagnostic integrity, (3) patient safety, and (4) staff safety. Each site independently assessed the transmission of the X‐ray beam through glass, the effect of the transmitted beam on image quality metrics, the effect of the transmitted beam on patient dose, and considerations of scattered radiation with the barrier present. Due to the independent data acquisition at the three institutions, not all measurements were collected from all three.

**Figure 1:**
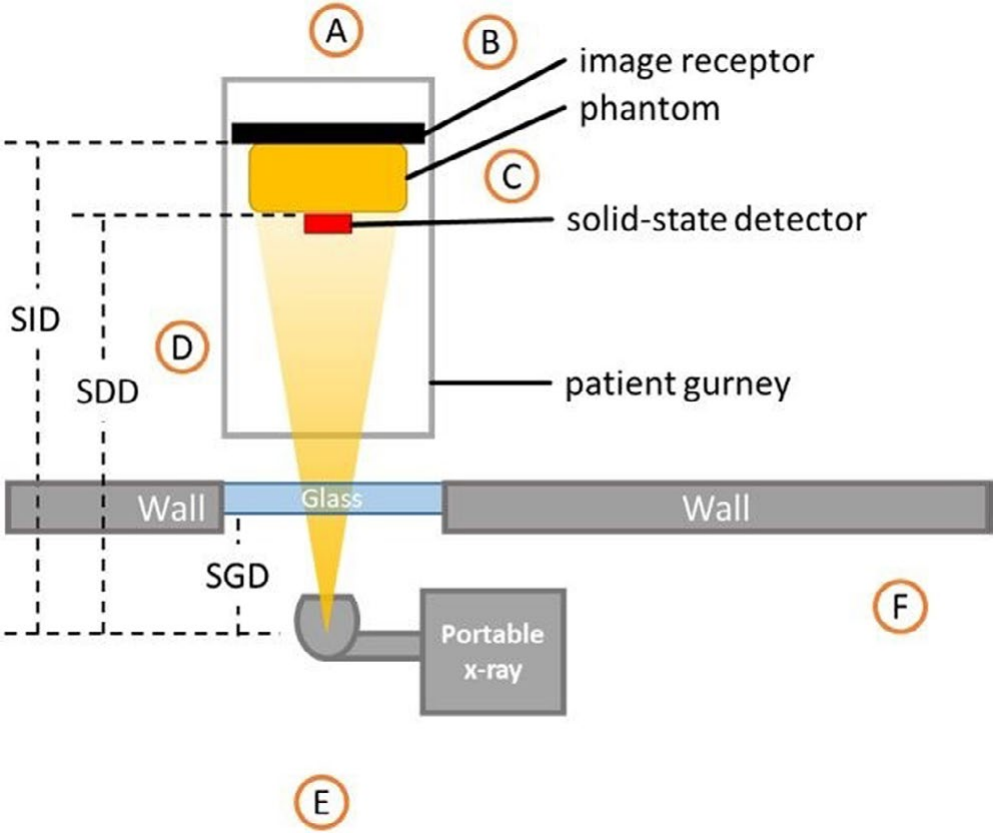
Experimental setup for transmission measurements. The source‐to‐image distance (SID), source‐to‐detector distance (SDD), and source‐to‐glass‐distance (SGD) differ by facility and are specified in Table 2

The experimental setup for Sections 2.1, 2.2, 2.3 is provided in Figure 1. At all sites, exposure was measured with a solid‐state radiation detector: at one site KPNC used a Raysafe X2 (Billdal, Sweden) and at two other sites KPNC used a Raysafe Xi. SCPMG used an RTI Piranha (Mölndal, Sweden), and SHC used a RadCal Accu‐Gold Multi‐Senor AGMS‐D+ (Monrovia, CA). Each site used different portable X‐ray imaging equipment: Canon RadPRO Mobile 3 (Irvine, CA) was used at KPNC and SCPMG, AGFA DX‐D100 (Mortsel, Belgium) was used at one site at SMC, and Carestream DRX (Rochester, NY) was used at another site at SMC. The image receptors for the systems were all digital (CsI) and calibrated to conform with the IEC 62494 specifications. The target index (TI) differed between the devices (Table 1).

**Table 1:** Acquisition parameters for beam transmission measurements with and without a glass barrier

Facility	Radiation detector	Portable radiographic unit	Image receptor	Target index (TI)	kV	Typical mAs	mAs adjustment for glass barriers	SDD (cm)	SID (cm)	SGD (cm)	Phantom
KPNC1	Raysafe Xi	RadPRO Mobile 3 & Canon DR	DR (CsI)	150	90	2	8	183	N/A		None used for transmission; *32 cm CTDI phantom used for scatter measurements*
KPNC2	Raysafe X2	RadPRO Mobile 3 & Canon DR	DR (CsI)	150	90	2	8	216	N/A		None
KPNC3	Raysafe Xi	RadPRO Mobile 3 & Canon DR	DR (CsI)	150	90	2	8	305	N/A		None used for transmission; *32 cm CTDI phantom used for scatter measurements*
SCPMG	RTI Piranha Black	RadPRO Mobile 3 & Canon DR	DR (CsI)	150	100	1.6	3.2	175	180	55	25 cm Chest Phantom; 5 cm acrylic + 3.2 mm Aluminum + 0.5 mm copper
SHC1	Accu‐Gold AGMS‐D+	AGFA DX‐D100	DR (CsI)	400	95	4	8	143	183	N/A	Anthropomorphic Chest (Kyoto Kagaku Lungman; Kyoto, Japan)
SHC2	Accu‐Gold AGMS‐D+	Carestream DRX Revolution	DR (CsI)	100	85	2.5	5	145	185	N/A	Anthropomorphic Chest (Kyoto Kagaku Lungman; Kyoto, Japan)

Additionally, a different phantom was used to evaluate image quality metrics: the CIRS 903 Fluoroscopy QA Phantom (Norfolk, VA) at SHC, the Pro Project Pro‐Fluo 150 Fluoroscopy QA Phantom (Okszow, Poland) at KPNC, and the Leeds TOR 18FG (North Yorkshire, UK) at SCPMG. Ultimately, only the phantoms at SHC and KPNC were analyzed quantitatively. Exposures were acquired with the glass barriers open and closed using the pre‐set technique for a typical AP chest examination. A patient chair or gurney was used to support the phantoms; the end of the gurney was typically within 30 cm of the door. Each site also acquired scattered radiation measurements around the patient and operator positions in this manner using an ion chamber (Section 2.4). A different phantom was used for scatter measurements at each institution. SCH used an anthropomorphic chest phantom, with an additional attenuating block to mimic a large patient, SCPMG also used an anthropomorphic chest phantom, and KPNC used the 32‐cm CTDI phantom, which is the standard for testing CT scatter of the torso.

### 2.1 Glass barrier transmission

Using the fitting parameters for plate glass published in NCRP 147,^8^ the expected transmission *B* of broad X‐ray beams through 0.635 cm (¼ʺ) of plate glass are shown in Appendix A and estimated by

(1) $$B={\left[\left(1+\frac{\beta }{\alpha }\right)e^{\alpha \gamma x}‐\frac{\beta }{\alpha }\right]}^{\frac{1}{\gamma }}$$

where *α*, *β*, and *γ* are fitting parameters and *x* represents the thickness of the barrier.

Glass barrier transmission measurements were performed with six radiographic systems at five facilities. The acquisition parameters are provided in Table 1 using the experimental setup in Figure 1.

### 2.2 Beam penetrability and diagnostic integrity

Tempered glass is primarily composed of silicon^9^ and acts as a beam‐hardening filter comparable to aluminum. Hardening was quantified with half‐value layer (HVL) measurements performed both with and without the glass door.

The exposure index (EI) and deviation index (DI) as reported by the imaging system were used as a surrogate for dose to the imaging receptor at the SCPMG and SHC sites. The EI is specific to each manufacturer and derived and calculated by proprietary methods and formulae. The DI is calculated by multiplying the base 10 log of the ratio of the measured EI and Target EI (TI) by 10. Images were acquired of a chest phantom (Table 1), with and without the glass barrier, using a fixed tube current and potential. The initial exposure technique selected without the glass barrier at all three institutions was the clinical default for a chest exam. The exposure time was increased with the glass barrier at SHC so that the DI was within the range −1 to 1 and at KPNC and SCMPG so that the entrance air kerma was matched. The resultant images were processed using a chest algorithm, and the exposure parameters are listed in Table 1.

**Figure 2:**
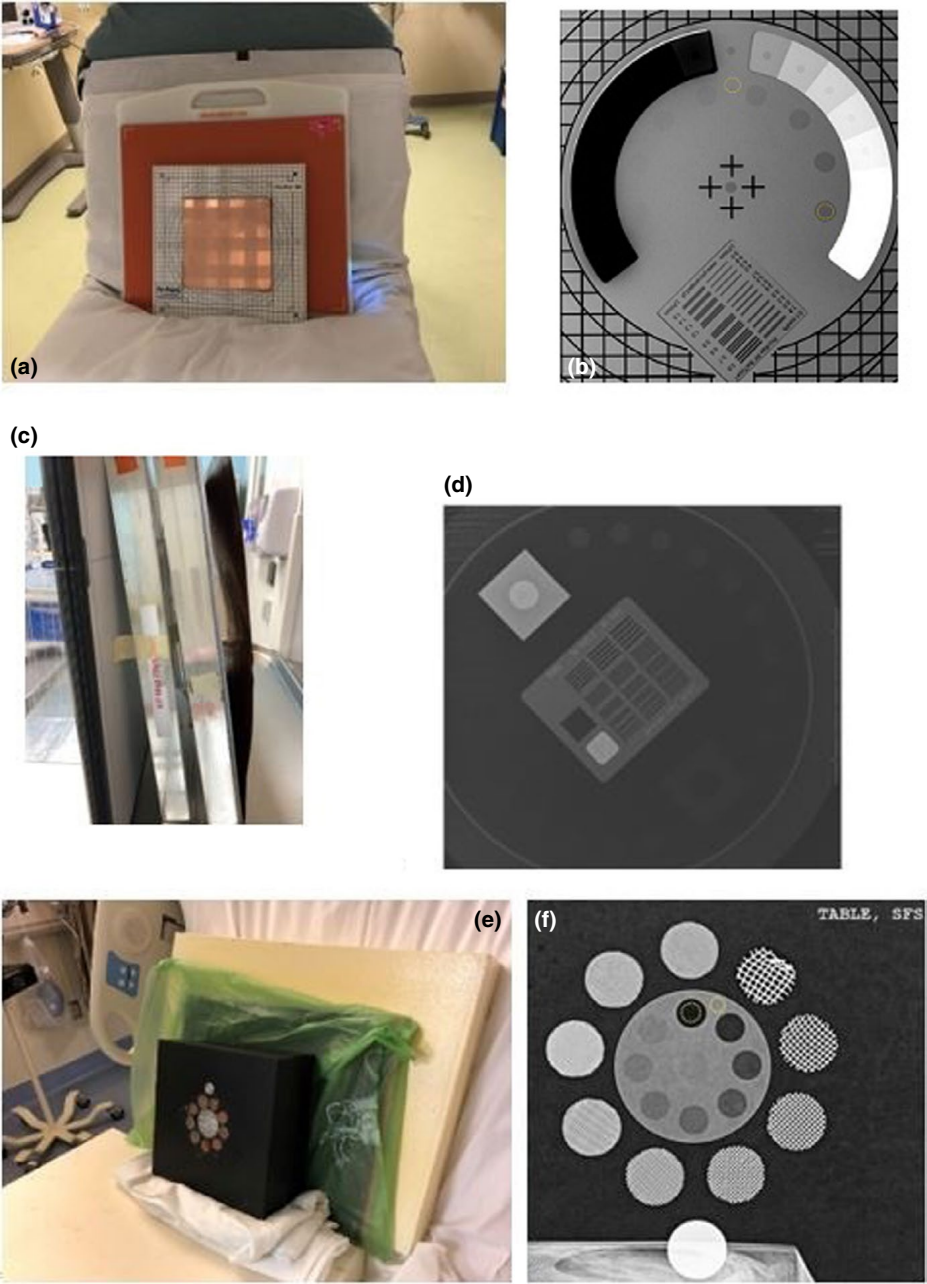
Experimental setup for image quality measurement. (Left) A photograph of the experimental set‐up with image quality phantoms at (a) KPNC, (c) SCMPG, and (e) SHC. (Right) The ROI placement for signal (dashed circle inside of the low contrast element) and background (dashed circle on the uniform background) at (b) KPNC and (f) SHC. The image quality phantom for SCPMG (d) is shown for reference

Exposures of image quality phantoms at all three institutions were acquired, as illustrated in Figure 2a–e. At two facilities, the contrast‐to‐noise ratio (CNR) was measured within each phantom, consisting of eight holes of varying depths. KPNC used ImageJ (National Institute of Health, New York, NY), and SHC used Intellispace Radiology 4.6 (Philips Healthcare, Amsterdam, Netherlands) to perform measurements. The signal was measured with a region of interest (ROI) placed inside each of the eight positions; the background and noise were measured from the uniform background as depicted in Figure 2b,f.

### 2.3 Patient safety

Each facility investigated patient exposure. Patient entrance air kerma (EAK) was estimated by a solid‐state radiation detector as illustrated in Figure 1. After determining the amount of transmission through the glass, additional exposures were acquired with a mAs setting to account for glass attenuation as described above; the updated mAs values are in Table 1 as “mAs adjustments for glass barriers.”

Patient effective dose was estimated for an adult using PCXMC Monte‐Carlo Imaging Software. The beam inherent filtration (in mm Al) was determined iteratively using SPEKTR 3.0 until the HVL of the beam matched that measured empirically. The filtration properties of the glass barrier were determined similarly from empirical HVL measurements, assuming a composition of 100% Si, and added to the inherent aluminum beam filtration. The default typical AP chest FOV provided by PCXMC was used. The software used the ICRP 103 methodology for estimating effective dose.

### 2.4 Staff safety

**Figure 3:**
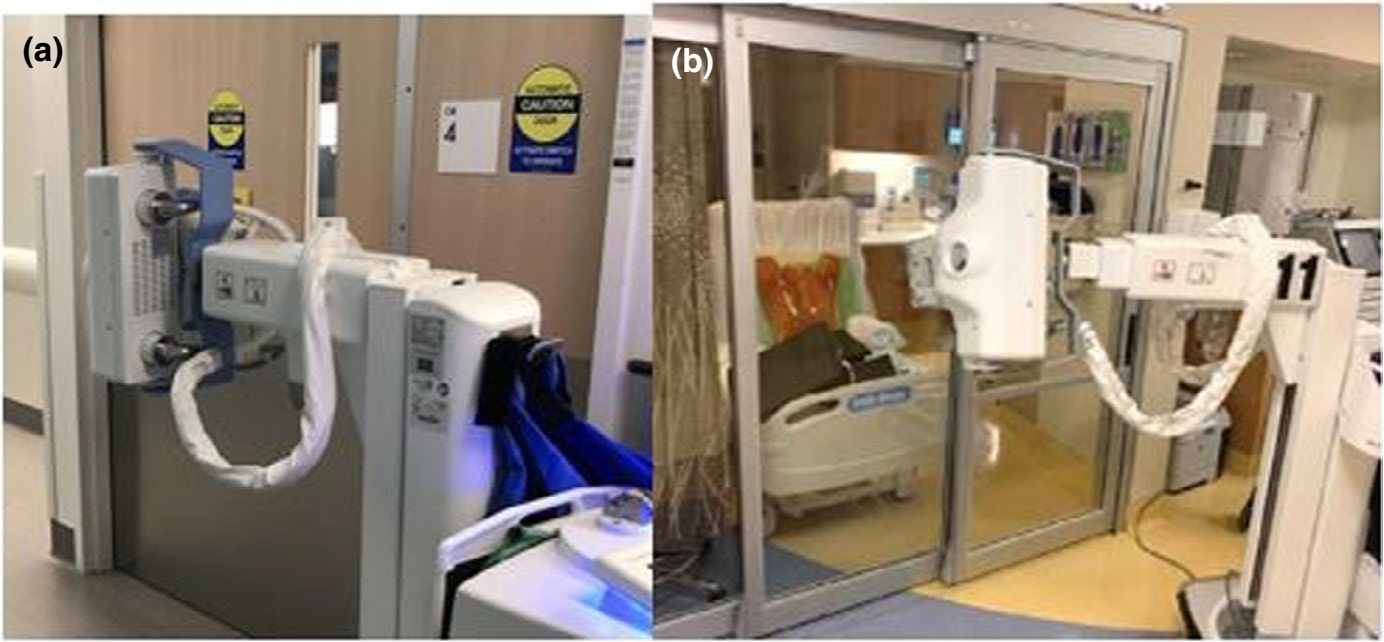
Phantom geometry for scatter measurements. Photographs of phantom geometry for scatter measurements at (a) SCPMG and (b) SHC. KPNC had a similar configuration as SHC except a 32 cm CTDI phantom was used as a scattering object

To measure scatter radiation exposure, the same basic geometry was used at each facility. A phantom was positioned on a gurney for a semi‐upright AP chest exposure, and the portable X‐ray unit was positioned outside of the patient room with the tube housing/collimator assembly placed adjacent to the glass (Figure 3). To quantify X‐ray scattering during patient imaging, each facility used a separate phantom as described above.

After adjusting the radiographic technique for decreased transmission through glass, each group independently measured scatter radiation exposure levels. To aid in comparability and synthesis of the scatter radiation measurements, a set of standardized measurement points was adopted, as illustrated in Figure 1. Due to variations in facility layouts, some measurement points could not be replicated at all facilities. Where possible and appropriate, inverse square law corrections were employed to normalize scatter radiation exposure levels to the standardized measurement points.

Safe levels of radiation exposure from scatter radiation for an “Uncontrolled Area,” an area requiring no additional shielding and able to be occupied by members of the general public, are defined as 0.02 mGy/week or 2.28 mR/week.^8^ Maximum workload was calculated by dividing the weekly air kerma limit or exposure level limits by the air kerma or exposure incurred at the standard locations from one portable chest X‐ray.

## 3 RESULTS

### 3.1 Glass barrier transmission

**Figure 4:**
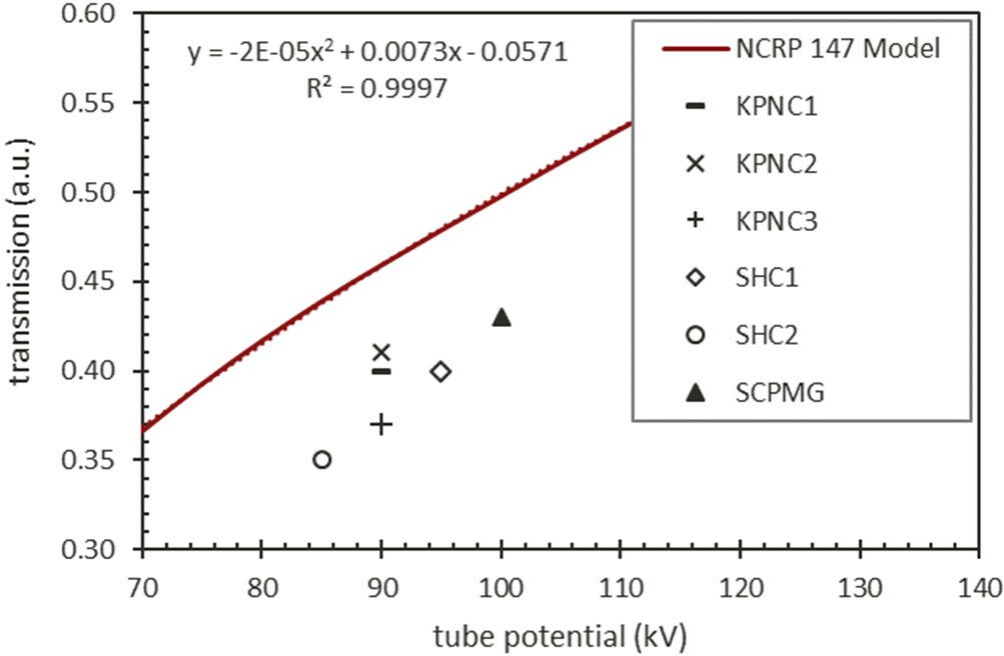
Transmission through barrier versus tube potential. The red line represents the transmission through glass relative to an unattenuated beam (in arbitrary units, a.u.) determined from Equation 1 as function of tube potential along with a polynomial fit. The points are the empirical transmission measurements

To characterize the scatter levels at the standardized measurement points for use of glass barriers, technique adjustments were incorporated, largely by increasing the technique by the reciprocal of the transmission values of the barriers. Figure 4 plots the values of those transmission values as measured by the independent groups, with one group measuring transmission values in three different facilities. There is a small (~4%) difference in transmission between KPNC1 and 2 versus KPNC3 that may be due to different glass thicknesses, as manufacturer and site specifications may vary, or slight variations in setup. All measurements fall below those estimated in the NCRP 147 model,^9^ possibly due to conservative estimations made in that report from a wide variety of glass sizes sampled.

With a mean transmission value of 39% through glass and standard deviation of 3%, an increase in technique by a factor of 2.5 would approximately yield an appropriate exposure level to the detector.

### 3.2 Beam penetrability and diagnostic integrity

Using a fixed technique, the EI with a glass barrier was less than the EI without the barrier (Table 2). While the transmission and EAK were reduced by 54% on average (Table 2), the average EI reduction was 39%. The reduction in transmission is consistent with measurements by others.^5^ Using EI as a surrogate for detector dose, these results indicate that 20% of the incident X‐ray spectrum is attenuated by the patient when there is no glass barrier. For SHC2, tube potential was held constant at 85 kV, while the mAs with the glass barrier was increased by a factor of 1.5 to match as closely as possible the EI without the glass barrier (EI = 217). The resulting mAs of 2.5 yielded an EI of 206. The DI values, which are a measure of the extent to which the EI varies from the target EI (TI), were −0.2 and −0.4 without and with the glass, respectively, indicating the EI had dropped slightly further from the target value with the glass but was still within a generally acceptable range (|DI| ≤ 1). The EAK also dropped from 34.32 uGy without glass to 19.39 uGy with glass, despite the increase in mAs. A more efficient, penetrating beam in the latter case is quantitatively evidenced by the relative beam hardness change, characterized by the half value layer (HVL) of aluminum. The HVL of the beam increased from 3.09 to 5.02 mm Al without and with glass, respectively, indicating fairly significant beam hardening by the glass barrier. At SCPMG, the tube potential was held constant at 100 kV, while integrated tube current was increased by a factor of 2 from 1.6 to 3.2 mAs to compensate for increased attenuation by the glass and to approximately match EAK. However, due to only certain mAs values being available to select on the portable, exact matching could not be performed. The twofold increase in integrated tube current in this case resulted in an EAK of 28.3 uGy, compared with 21.0 uGy yielded by the technique without the glass, a 35% increase in EAK. The resulting EIs were 26 and 69, and the DIs were −7.66 and −3.55 without and with glass, respectively. It was observed by the SCPMG that the TI values had not been adjusted for the phantom used, resulting in the very large‐magnitude DI. The HVL increased from 3.13 to 5.1 mm Al without and with glass, respectively, again indicating fairly significant beam hardening by the glass barrier. The results of SHC2 demonstrate a 44% decrease in EAK when EI matching was used. The results of SCPMG demonstrate an increase of EI of 165% when EAK matching was used. Again, perfect matching could not be performed due to the discrete nature of mAs steps available on the portable units. The HVL with glass was similar to those found by others.^5^

**Table 2:** Radiation measurements and patient effective dose estimates with and without a glass barrier

Technique	Make	kVp	mAs	mA	ms	Without Glass					With Glass					%EAK Reduction	%EI Reduction
						EI	DI	EAK (uGy)	HVL (mmAl)	Effective dose (µSv)^a^	EI	DI	EAK (uGy)	HVL (mmAl)	Effective dose (µSv)^a^		
Typical	KPNC1	90	2					31.4					12.6			59.9	
Typical	KPNC2	90	2					30.3					12.4			59.1	
Typical	KPNC3	90	2					22.5					8.4			62.7	
Typical	SCPMG	100	1.6	320	5	34^b^	−7.7	21.0	3.23	7.2	26^c^	—	9.1	4.95	3.9	56.7	23.5
*Adjusted for glass*	SCPMG	100	3.2	400	8	69^c^	—	44.1	—	14.4^d^	51^c^	−3.4	28.3	5.09	7.7^d^	35.8	26.1
Typical	SHC1	95	4	400	10	442	0.4	57.8	3.65	21.0	282	−1.5	23.01	5.66	10.0	60.2	36.2
*Adjusted for glass*	SHC1	95	6.4	400	16	—	—	—	—	33.3^d^	458	0.6	37.28	5.74	16.6^d^	—	—
Typical	SHC2	85	1.6	320	5	217	−0.2	34.32	3.09	11.5^d^	—	—	—	—	5.3^d^	—	—
*Adjusted for glass*	SHC2	85	2.5	312	8	358	3.0	55.23	3.14	18.0	206	−0.4	19.39	5.20	8.3	64.9	42.5

Note

Only EAK data were collected from KPNC.

^a^ A value obtained with a PCXMC simulation.

^b^ A value estimated iteratively with SPEKTR 3.0.

^c^ Exposure index for Canon system is calculated using a manufacturer‐specific proprietary method that varies linearly with exposure.

^d^ The value was calculated from system mAs and mSv/mAs.

**Figure 5:**
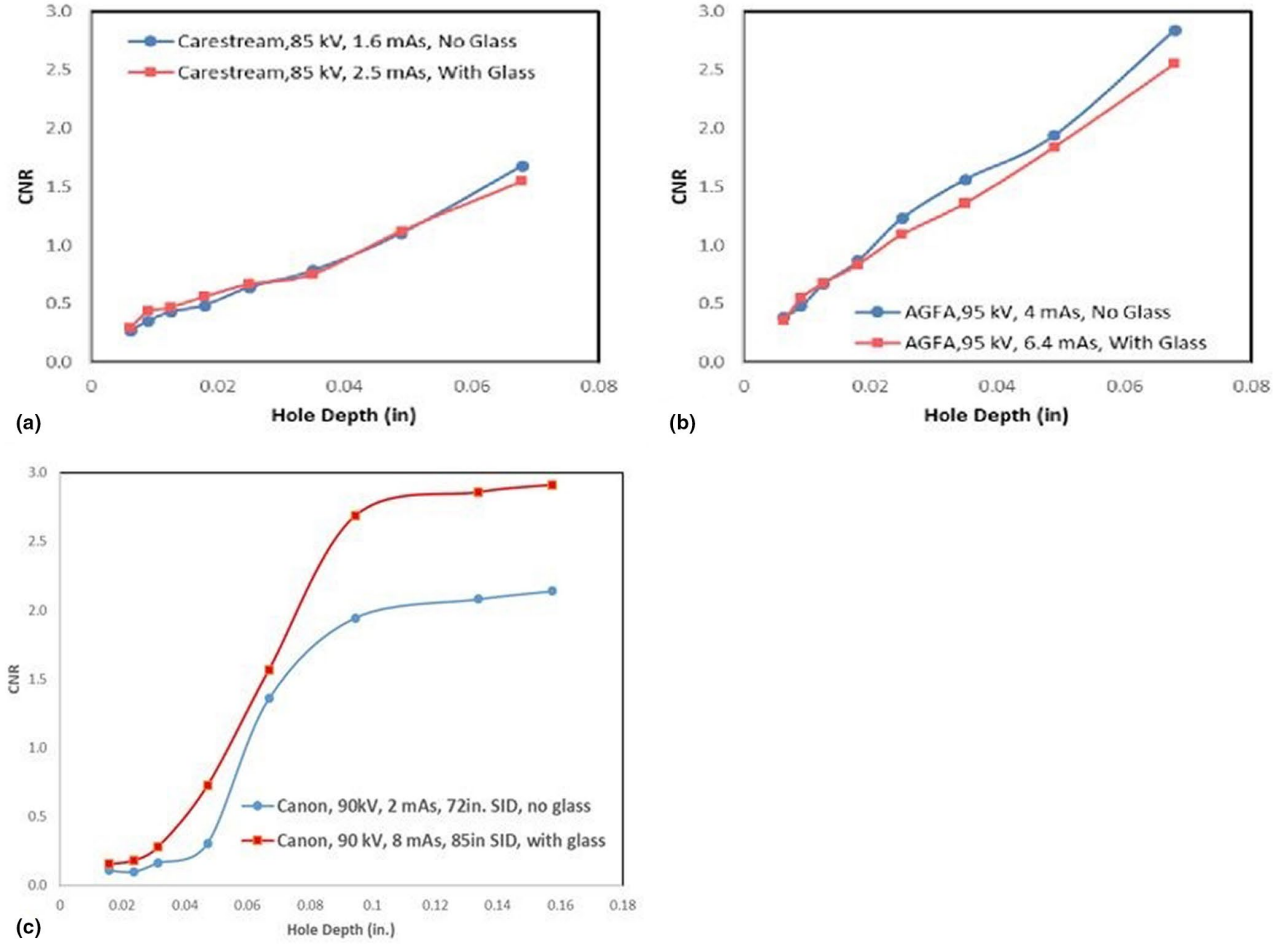
CNR as a function of low‐resolution phantom hole depth. CNR as a function of hole depth within the low contrast resolution module of the CIRS phantom for SHC2 (a), SHC1 (b), and the Pro Fluo‐150 phantom for KPNC3 (c) portable X‐ray systems. For each system, the CNR was measured under normal beam conditions (blue) and with the glass door closed (red)

The CNR performance is not comparable across all eight models of portable systems. The low contrast resolution is highly dependent on image processing parameters as well as the use of grids. Within this study, none of the institutions regularly employed grids with portable imaging. However, the image processing was not uniformly applied between institutions. The results in Figure 5 indicate that the CNR was comparable (SHC1 and SCH2) or improved (KPNC2) with the glass barrier. In the case of KPNC3, the CNR may have been improved by reduced image noise from the increased mAs used with glass. These results suggest that imaging performance with the glass is acceptable for low‐contrast diagnostic tasks.

### 3.3 Patient safety

The reduction in effective dose with the glass barrier ranged from 50% to 80% for the same technique (Table 2), presumably due to a decreased entrance air kerma and a more penetrating beam. When an increase in technique was taken into account to normalize the exposure to the detector when imaging through glass at the same SID (Appendix A), the estimated effective dose to the patient increased for the three techniques increased by about 5%–10% (Table 3). The increase is of the magnitude of 1 µSv, or 0.03% of the average annual exposure to background radiation in the United States.^10^ Actual changes to effective dose will depend on the available kV/mAs selections on the machine, which are not taken into account here.

**Table 3:** Estimated effective dose changes to patient with and without glass barrier

Make	kVp	Typical mAs/mAs × f_glass, kV_	Effective Dose (µSv)		% change
			At typical mAs without glass	At mAs × f_glass, kV_ with Glass	
SCPMG	100	1.6/3.2	7.2	7.7	7%
SHC1	95	4/8.4	20.8	21.7	4%
SHC2	85	1.6/3.8	11.5	12.7	10%

### 3.4 Staff safety

After the technique adjustments were ascertained, the phantoms were exposed through the glass barriers with the techniques adjusted for glass (Table 2). The individual scatter air kerma (exposure) measurements that corresponded to the positions shown in Figure 1 for the individual groups are shown in Table 4, using the same setups and techniques as Table 1. Table 4 also gives the mean air kerma (exposure) measurements, as well as the uncertainties in those values. KPNC1 and KPNC2 had similar layouts and glass barriers, so the data were only acquired at one of the facilities. The magnitude of the air kerma measured is similar to those demonstrated by others, and highest scatter being measured at the 45° location (Position D in Figure 1) is consistent with previous investigators.^5^

**Table 4:** The air kerma* values at the standardized measurement points are shown below

Location	SHC3	KPNC1	SCPMG	KPNC3	Mean	St Dev	Allowable number of weekly PCXRs
1 m behind patient (A)	7.9 ± 0.8			155 ± 16	82	105	245
1 m diag forward scatter (B)	141 ± 14	179 ± 18		190 ± 19	170	26	117
1 m side of patient (C)	147 ± 47	261 ± 26	86 ± 9	212 ± 21	177	76	113
1 m 45° backscatter (D)	141 ± 14	261 ± 26		276 ± 28	226	74	88
Operator 1 m from tube behind glass (E)	19 ± 2	87 ± 9		259 ± 26	122	124	164
~ 3 m from tube (F)	3.5 ± 0.3			4.3 ± 0.4	3.9	0.6	5,101
	* Air Kerma (nGy) (1 nGy = .114 uR exposure)						

Note

A conservative calibration error of 10% has been applied.

Table 4 details the composite average exposures as well as the number of allowable portable chest X‐rays in which staff can engage and still be considered “safe” as defined by NCRP 147's Uncontrolled Area limit. Although these numbers are considered safe for the staff and general public, appropriate shielding materials including lead (equivalent) aprons and mobile shields should be used to keep doses as low as reasonably achievable.

## 4 DISCUSSION

This work presents an evaluation of image through glass in terms of transmission, image quality, patient safety, and staff safety performed by three independent groups. The glass barriers attenuated a mean of 61% of the incident X‐ray beams, necessitating approximately 2.5 times increase in beam intensity. Further refinements were made for increased SID and beam quality to adequately match EI values as reported by the image receptors.

When the mAs was increased to account for transmission reductions, the CNR with glass was equivalent or higher than without glass. While matching EI resulted in comparable CNR measurements at SHC, during clinical review, the noise levels were deemed unacceptable, particularly for large patients, and a mAs increase of two times the standard protocol for all patient sizes was implemented.

For staff safety, it is advantageous to position the base unit of the portable X‐ray system such that it shields the operator and bystanders. Position D had the highest exposure reading, due to backscattering from the phantom and forward scattering from the glass. It is recommended that any staff member that must be in the room stands in position A, B, or C.

### 4.1 Fast deployment recommendations

Given the time‐sensitive nature of establishing a surge area in a pandemic, fast deployment is a key component of implementing the strategies described above for portable radiographic imaging. In addition to the considerations for patient radiation exposure and image quality, a quick assessment must be made of radiation exposure for staff and members of the public.

Consider the following to aid in fast deployment.

1. Determine the medical center's patient workload needs for use of portable imaging with clinicians.
2. Take radiation exposure measurements using a calibrated survey meter at multiple locations around the portable X‐ray unit and patient to make shielding recommendations.
3. Determine a maximum number of acquisitions to be performed per week without shielding present or specifying the amount of shielding needed for the desired maximum possible workload.
4. Consider local, state, and federal regulations that must be met. Seek guidance from regulators when special circumstances arise.
5. In the case of imaging through a new barrier without a survey meter, if direct measurement is not feasible, local staff can estimate transmission using the relative EI reported by a digital image receptor with and without the barrier. While the actual EI performance compared to the expected values are varied, the relative comparison should yield enough information about transmission.

This procedure is suggested as a starting point for technique modification. From there, monitor the EI values in the resulting radiographs, so that fine‐tuning may be performed to maintain image quality. It is suggested that a trial period of at least 1 week be conducted with a daily radiologist review and iterative fine‐tuning. It is best to limit imaging to just a few technologists and portable X‐ray machines during the trial period. In fact, at one institution, the implemented program continues to include a daily quality review of COVID portable X‐ray performed by a limited group of technologists and portables.

### 4.2 Limitations

There were several limitations and sources of variability within this study. This study is limited by the number of glass samples and tube potentials investigated. Because this work was performed independently, patient dose estimates are not available for all sites.

Because each facility independently evaluated imaging through glass barriers, the acquisition methods are neither standardized nor performed at each site. For ease of interpretation, Table 5 summarizes the evaluations performed at each site. This work demonstrated the need for a more standard method of evaluating CNR. Each facility employed a different phantom. Additionally, image processing is specific to anatomic region, vendor, and even variable by machine. Image processing was not applied consistently between systems; consequently, the slope of the CNR curves for different systems varies considerably. Additional measurements to determine the magnitude of error would be ideal in a subsequent analysis.

**Table 5:** Summary of contributing facilities to each type of evaluation

	Evaluation	Glass Barrier Transmission	Beam Penetrability and Diagnostic Integrity		Patient safety	Staff safety
	*Metric*	*Entrance Air Kerma*	*Exposure Index*	*Contrast‐to‐Noise Ratio*	*Effective Dose*	*Scatter*
Facility	KPNC1	✓	✓			✓
	KPNC2	✓				
	KPNC3	✓		✓		✓
	SCPMG	✓	✓		✓	✓
	SHC1	✓	✓	✓	✓	
	SHC2	✓	✓	✓	✓	✓

Note

The metric used for the evaluation is italicized in the second row.

As Table 4 shows, there is significant uncertainty in the scatter measurements both behind the patient and behind the X‐ray unit. In the former case, uncertainties are from differences in the photon absorption properties of the detector and patient bed. In the later, uncertainties arise from the absorption of scattered photons by the tube/collimator assembly and/or portable base. Additional transmission measurements would be needed to determine the degree of uncertainty.

Another limitation is that a formal clinical evaluation of images was not performed in this work. A retrospective quality review of images acquired through barriers was conducted with radiologists at each respective medical center. The radiologists surveyed indicated that all images were diagnostically acceptable with minimal differences observed from examinations acquired without a glass barrier in place once a two times mAs practice was widely implemented.

## 5 CONCLUSIONS

In this work, the three independent groups were able to answer questions regarding patient and staff safety, image quality, and requisite technique adjustments when performing portable chest X‐rays through glass barriers. Given the adequate technique adjustments, there was no discernible degradation of image quality as determined by objective CNR measurements and corroborated by radiologist assessments. The glass‐hardened beams resulted in essentially unchanged patient doses. Scatter from both the phantoms and the glass barriers themselves resulted in relatively low exposure levels. That said, protective measures such as the use of lead aprons, mobile shields, and increased distance from the scattering sources should be employed where practicable.

## CONFLICT OF INTEREST

The authors declare no conflict of interest.

## AUTHOR CONTRIBUTIONS

Sarah E. McKenney coordinated the project, and provided data collection, data analysis, manuscript editing. John M. S. Wait provided data collection, data analysis, manuscript editing, and manuscript submission. Virgil N. Cooper, III provided data collection, data analysis, and manuscript editing. Amirh M. Johnson provided data collection, data analysis, and manuscript editing. Jia Wang provided data collection, and manuscript editing. Ann N. Leung provided data review and manuscript editing. Jessica Clements conceived the collaboration and also provided data collection and manuscript editing.

## DATA AVAILABILITY STATEMENT

The data that support the findings of this study are available from the corresponding author upon reasonable request.

## Figures and Tables

**Figure A1:**
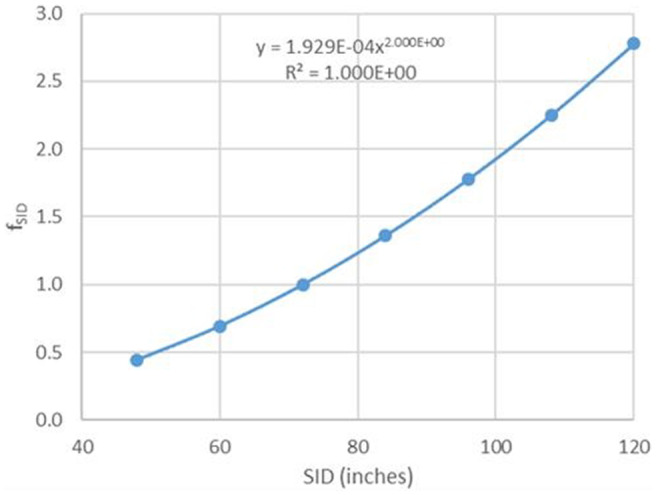
Technique adjustment factor for SID over SID. The blue line represents the f_SID_ factor, that is, the mAs compensation for increasing or decreasing the SID from 72 in

## Appendix A

The typical conventional portable chest radiograph is acquired at a 72‐in. SID with no additional glass barrier interposed between the X‐ray tube/collimator assembly and the patient. In portable chest radiography through glass barriers, the addition of the glass barriers reduces the photon fluence to the image receptor, so compensatory adjustments must be made to the radiographic technique. Also, the SID may need to increase due to physical limitations imposed by the patient room layout, thus requiring additional inverse square law corrections to maintain appropriate fluence to the imaging receptor. Below is an equation to modify existing technique factors to account for the glass barrier and increased SID to ensure similar fluence to the imaging receptor. The f_glass, kV_ factor (Table A1) accounts for the beam attenuation of the glass as a function of tube potential (kV).^8^ The f_SID_ factor (Figure A1) accounts for changes in the SID. Bear in mind that these factors may result in many‐fold increases in technique due to photon attenuation and inverse square law effects.

(2) $${\text{mAs}}_{\text{new}}={\text{mAs}}_{\text{old}}\times f_{\text{glass},\;\text{kV}}\times f_{\text{SID}}$$

### *Example*:

A facility typically performs its portable chest X‐rays at 95 kV and 3 mAs with a SID of 72 in. With the bed and tube as close to the glass as possible, the SID increases by one foot to 84 in. What should the new mAs be?

$${\text{mAs}}_{\text{new}}=\text{3 mAs}\times 2.09\times 1.36$$

$${\text{mAs}}_{\text{new}}=8.5\;\text{mAs}$$

**Table A1:** Scaling factors to account for transmission reduction from 0.635 cm (¼ʺ) plate glass

kV	*f_glass_*_, kV_
70	2.7
75	2.5
80	2.4
85	2.2
90	2.2
95	2.1
100	2.0
105	1.9
110	1.9
115	1.8
120	1.7

## References

[1] Auffermann WF, Kraft CS, Vanairsdale S, Lyon GM, Tridandapani S. Radiographic imaging for patients with contagious infectious diseases: how to acquire chest radiographs of patients infected with the Ebola virus. Am J Roentgenol. 2014;204(1):44‐48. 10.2214/AJR.14.14041 25402496

[2] Rubin GD, Ryerson CJ, Haramati LB, et al. The role of chest imaging in patient management during the COVID‐19 pandemic: a multinational consensus statement from the Fleischner Society. Radiology. 2020;296(1):172‐180. 32255413 10.1148/radiol.2020201365 PMC7233395

[3] Mossa‐Basha M, Medverd J, Linnau K, et al. Policies and guidelines for COVID‐19 preparedness: experiences from the University of Washington. Radiology. 2020;296(2):E26‐E31. 10.1148/radiol.2019201326 32687455

[4] Mozdy M. Safer, PPE‐Conserving X‐Rays for Patients at University Hospital | University of Utah. https://medicine.utah.edu/radiology/news/2020/04/x‐ray‐through‐glass.php. Accessed June 11, 2020.

[5] Brady Z, Scoullar H, Grinsted B, et al. Technique, radiation safety and image quality for chest X‐ray imaging through glass and in mobile settings during the COVID‐19 pandemic. Phys Eng Sci Med. 2020;43:765‐779. 32662037 10.1007/s13246-020-00899-8 PMC7355508

[6] Moirano JM, Dunnam JS, Zamora DA, et al. Through the glass portable radiography of patients in isolation units: experience during the coronavirus disease (COVID‐19) pandemic. Am J Roentgenol. 2020; 10.2214/AJR/2-/23367. 33236649

[7] Liu TY, Rai A, Ditkofsky N, et al. Cost benefit analysis of portable chest radiography through glass: initial experience at a tertiary care center during COVID‐19 pandemic. J Med Imaging Radiat Sci. 2021; 10.1016/j.jmir.2021.03.036. PMC8026266 33875400

[8] NCRP National Council on Radiation Protection and Measurements . Structural shielding design for medical x‐ray imaging facilities. NCRP Report No. 147, National Council on Radiation Protection and Measurements, Bethesda, Maryland, 2004.

[9] Code of Federal Regulations . Safety Standard for Architectural Glazing Materials. 16 CFR 1201.

[10] NCRP National Council on Radiation Protection and Measurements . Ionizing Radiation Exposure of the Population of the United States. NCRP Report No. 160, National Council on Radiation Protection and Measurements, Bethesda, Maryland, 2009.

